# Preconditioning of Bioactive Glasses before Introduction to Static Cell Culture: What Is Really Necessary?

**DOI:** 10.3390/mps3020038

**Published:** 2020-05-09

**Authors:** Frederike Hohenbild, Marcela Arango-Ospina, Arash Moghaddam, Aldo R. Boccaccini, Fabian Westhauser

**Affiliations:** 1 Center of Orthopedics, Traumatology and Spinal Cord Injury, Heidelberg University Hospital, Schlierbacher Landstraße 200a, 69118 Heidelberg, Germany; Frederike.Hohenbild@med.uni-heidelberg.de; 2 Institute of Biomaterials, University of Erlangen-Nuremberg, Cauerstraße 6, 91058 Erlangen, Germany; Marcela.Arango@fau.de (M.A.-O.); Aldo.Boccaccini@ww.uni-erlangen.de (A.R.B.); 3 ATORG—Aschaffenburg Trauma and Orthopedic Research Group, Center for Trauma Surgery, Orthopedics, and Sports Medicine, Klinikum Aschaffenburg-Alzenau, Am Hasenkopf 1, 63739 Aschaffenburg, Germany; Arash.Moghaddam-Alvandi@klinikum-ab-alz.de

**Keywords:** 45S5 bioactive glass, biocompatibility, passivation, cytocompatibility, cytotoxicity, human bone marrow-derived mesenchymal stromal cells

## Abstract

Due to their high bioreactivity, the in-vitro analysis of bioactive glasses (BGs) can be challenging when it comes to maintaining a physiological pH. To improve BG biocompatibility, a heterogenic spectrum of preconditioning approaches, such as “passivation” of the BGs by incubation in cell culture medium, are used but have never been directly compared. In this study, the effect of passivation periods of up to 72 h on pH alkalization and viability of human bone marrow-derived mesenchymal stromal cells was evaluated to determine a time-efficient passivation protocol using granules based on the 45S5-BG composition (in wt%: 45.0 SiO_2_, 24.5 Na_2_O, 24.5 CaO, 6.0 P_2_O_5_) in different concentrations. pH alkalization was most reduced after passivation of 24 h. Cell viability continuously improved with increasing passivation time being significantly higher after passivation of at least 24 h compared to non-passivated 45S5-BG and the necessary passivation time increased with increasing BG concentrations. In this setting, a passivation period of 24 h presented as an effective approach to provide a biocompatible cell culture setting. In conclusion, before introduction of BGs in cell culture, different passivation periods should be evaluated in order to meet the respective experimental settings, e.g., by following the experimental protocols used in this study.

## 1. Introduction

Since the introduction of the 45S5-bioactive glass (BG) composition (in wt%: 45.0 SiO_2_, 24.5 Na_2_O, 24.5 CaO, 6.0 P_2_O_5_) by Hench and coworkers in the late 1960s, the family of BGs grew rapidly [1,2]. Since then, various types of BGs have been investigated in different fields of research including, but not limited to bone tissue engineering (BTE) [3,4]. For application in BTE, BGs are particularly attractive due to the changes on their surface when in contact with physiological fluids that lead to strong bonding to surrounding tissues [5,6]. Furthermore, the release of bioactive ions like silica from the BGs is known to stimulate osteogenic differentiation of bone precursor cells and the formation and calcification of the osseous extracellular matrix [7,8].

When new BG compositions are introduced to BTE applications, it is of particular interest to investigate their biological properties in-vitro, including cytotoxicity assays or their impact on osteogenic differentiation [8,9]. As per definition, BGs exhibit a high surface reactivity, allowing their surface to remodel in contact to fluids due to the following cascade of reactions: Within minutes a rapid exchange of alkali ions with hydrogen (H^+^ or H_3_O^+^) occurs, leaving a poorly connected silica network and consecutively leading to the formation of a silica-rich surface layer. Calcium hydroxyl phosphate precipitates and crystallization leads to the formation of a hydroxycarbonate apatite layer. This layer is capable of incorporating organic components and allows living bone to bond as it is highly similar to bone mineral [10,11,12].

However, the described burst release of alkali ions leads to a strong increase in local pH potentially resulting in pH-dependent cytotoxicity [13,14,15,16]. Apart from a compromise of cell viability, rapid pH changes in cell culture medium can confound the interpretation of cellular function [17]. Therefore, when introduced to cell culture studies, BGs have to be prepared using so-called preconditioning procedures such as “passivation” by introducing the BGs to cell culture medium for a certain period of time before allowing contact to cells [8,18] to prevent the cells from the initial burst release of ions and its consequences.

Preconditioning limits the BG-mediated pH changes to a significant extent and does not alter the hydroxycarbonate apatite (HCA) formation ability as demonstrated by Pryce and co-workers [19]. In a review conducted by our group, different preconditioning approaches were analyzed showing a broad variety in the used methods [8]. For example, Lin et al. pre-incubated BG-based scaffolds for 1 h before introduction to a cell culture setting [20] whilst Detsch and co-workers preconditioned 45S5-BG based scaffolds with comparable porosity for 2 weeks before cell seeding [21].

The broad spectrum of passivation protocols might cause confusion when it comes to the planning of BG-based cell culture studies. Certainly, preconditioning of BGs prior to use in cell culture settings is important, however, it remains unclear how different passivation periods actually influence cell viability and growth in one and the same setting.

Therefore, in this study, granules made from 45S5-BG in three different concentrations were introduced to a culture of human bone marrow-derived mesenchymal stromal cells (BMSCs) without and with passivation in Dulbecco’s Modified Eagle’s Medium (DMEM) for 1, 6, 24, and 72 h. The impact of the different preconditioning periods on cell viability and growth patterns as well as their impact on the pH of the cell culture medium (CCM) was assessed and compared to a control consisting of BMSCs in BG-free CCM. This work will help researchers to plan cell culture experiments involving BGs more accurately in a time-effective manner.

## 2. Materials and Methods

### 2.1. General Experimental Design: Overview

**Figure 1:**
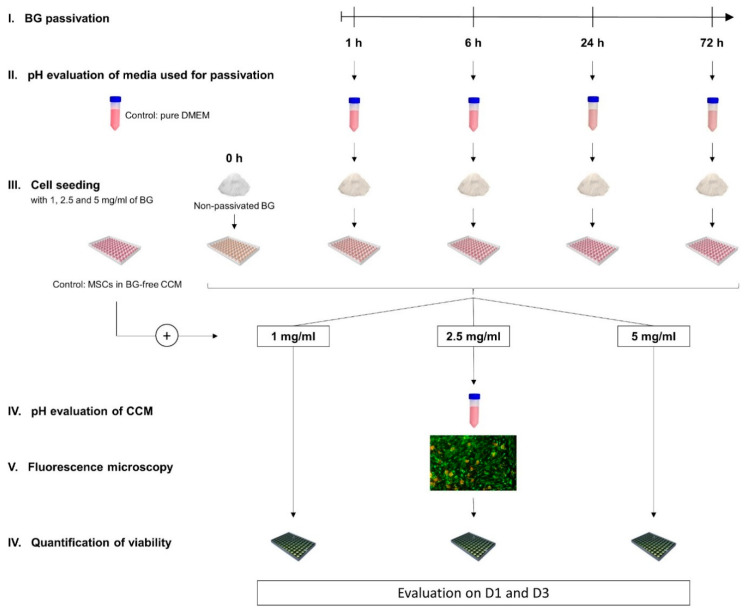
Schematic overview of the experimental design. Bioactive glass (BG) granules were passivated for 1, 6, 24, and 72 h respectively. pH values of the Dulbecco’s Modified Eagle’s Medium (DMEM) used for passivation were measured. Passivated BGs and non-passivated BGs (0 h) at different concentrations were co-cultured with human bone marrow-derived mesenchymal stromal cells (BMSCs) while cells in BG-free cell culture medium (CCM) served as control. On day 1 (D1) and day 3 (D3) the average BG concentration was used for pH evaluation and microscopical analysis of cell growth patterns, whereas a quantification of cell viability in all groups was performed at BG concentrations of 1, 2.5, and 5 mg/mL.

45S5-BG granules were subjected to dynamic passivation in DMEM high glucose (Life Technologies, Darmstadt, Germany) for 1 h, 6 h, 24 h, and 72 h respectively. After the designated period of passivation, pH values of the media utilized for the passivation process were analyzed using pure DMEM as control. Conditioned medium was discarded and passivated BG granules as well as non-passivated BG granules (0 h) were added to fresh CCM (89% DMEM high glucose, 10% fetal calf serum (FCS; Life Technologies, Darmstadt, Germany), 1% penicillin/streptomycin (Biochrom, Berlin, Germany)). Medium containing the BG granules was placed in 96-well cell culture plates (Nunc, Roskilde, Denmark) and BMSCs were added achieving a cell density of 2 × 10^4^ cells per cm^2^ and final BG concentrations of 1, 2.5, and 5 mg/mL. A control group was composed of BMSCs in BG-free CCM while each group consisted of 5 biological replicates. After 1 (D1) and 3 (D3) days of incubation the middle concentration of 2.5 mg/mL was used to evaluate pH values in the CCM and microscopically analyze cell morphology and growth patterns in direct presence of the differently preconditioned BGs. To quantify the influence on cell viability a fluorescence-based assay was performed for all groups as schematically depicted in Figure 1.

### 2.2. BG Production and Characterization

**Figure 2:**
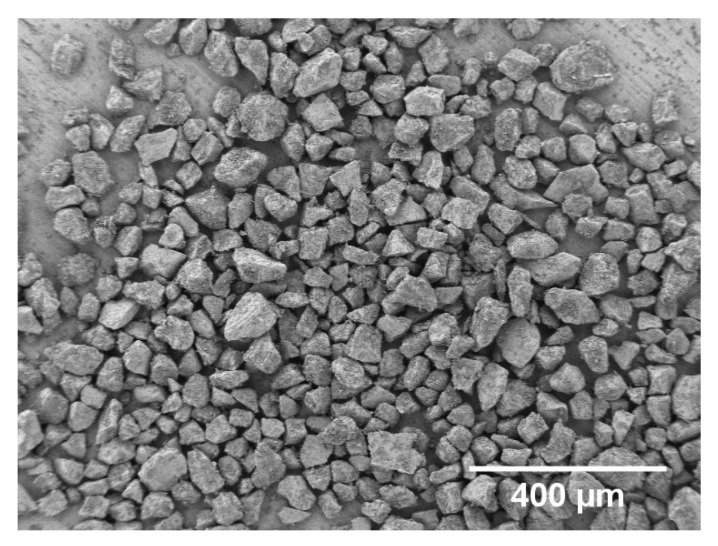
Scanning electron microscopy (SEM) image of the investigated bioactive glass (BG) granules prior to passivation depicted at a 200-fold magnification. The image was taken using a secondary electron detector, at a voltage of 1.5 kV and working distance of 5.1 mm. The reference bar refers to 400 µm.

45S5-BG was prepared following the melt-quench route using analytical grade reagents, namely, NaCO_3_ (Honeywell Fluka, Steinheim, Germany), CaCO_3_ (Honeywell Fluka, Steinheim, Germany), CaHPO_4_·2H_2_O (Acros Organics, Geel, Belgium), and commercial grade Belgian quartz sand (SiO_2_). The reagents were mixed together in a platinum crucible, heated up to 1400 °C for 1 h and quenched rapidly in water. The glass frits were crushed with a Jaw Crusher (Retsch, Haan, Germany) and ground into fine powder with a planetary ball mill (Retsch, Haan, Germany) with absolute ethanol (VWR Chemicals, Radnor, PA, USA) as milling medium. The powder was subsequently sintered at 1050 °C for 2 h, ground and sieved to obtain particles less than 125 µm. The morphology of the final granules was characterized by a polygonal shape with average size of 66 ± 19 µm as depicted in Figure 2 (SEM Auriga, Carl-Zeiss, Jena, Germany). The average size was estimated from scanning electron microscopy (SEM) images using the software ImageJ (National Institutes of Health Bethesda, MD, USA).

### 2.3. Passivation of the BG-Particles

In a heating and drying oven (Heraeus Instruments, Hanau, Germany) 45S5-BG granules were sterilized at 160 °C for 30 min. During the sterilization period, the 45S5-BG granules were stored in a glass beaker that was airtight sealed with aluminum foil. The sterilized BG granules were then added to DMEM high glucose at a concentration of 5 mg/mL and transferred to 50 mL falcon tubes (Merck, Darmstadt, Germany). On an orbital shaker (neoLab, Heidelberg, Germany) the BG was incubated at 37 °C and 5% CO_2_ at 100 rpm. After 1, 6, 24, and 72 h the falcon tubes were centrifuged at 10,000 rpm for 10 min; supernatant was removed and used for pH evaluation. BG-granules were kept dry until added to CCM at concentrations of 1, 2.5, and 5 mg/mL.

### 2.4. Study Ethics and Cell Origin

BMSCs of a 20-year-old male patient undergoing surgery at the proximal femur at the Heidelberg University Hospital were harvested. Prior to cell collection, written consent was obtained from the patient and the responsible ethics committee of the Medical Faculty of Heidelberg University approved the study (S-340/2018).

### 2.5. BMSC Isolation, Cultivation, and Characterization

BMSCs were isolated from bone marrow washouts as described previously [22,23,24]. In short, bone marrow was collected in a heparinized syringe and cells were fractioned on a Ficoll-Paque Plus density gradient (GE Healthcare Europe, Freiburg, Germany). The fraction of mononuclear cells containing the BMSCs was washed in PBS (Life Technologies, Darmstadt, Germany) and seeded in several 0.1% gelatin-coated (Sigma-Aldrich, Steinheim, Germany) T75 cell culture flasks (Nunc, Roskilde, Denmark).

Cells were cultivated in expansion medium composed of DMEM high glucose supplemented with 12.5% FCS, 1% penicillin/streptomycin, 1% L-glutamine (Life Technologies, Darmstadt, Germany), 1% non-essential amino acids (NEAA; Life Technologies, Darmstadt, Germany), 0.1% β-mercaptoethanol (Life Technologies, Darmstadt, Germany) and 4 ng/mL fibroblast growth factor 2 (Abcam, Cambridge, U.K.). To discard non-adherent cells, medium was changed after the first 24 h and subsequently twice weekly. Cells were passaged at 80% confluency and stored in liquid nitrogen until further use in passage 4.

### 2.6. Evaluation of pH

Prior to the start of the incubation, pH values of the medium used for preconditioning of the BG were measured. The conditioned medium, as well as pure DMEM serving as control, were transferred to falcon tubes, which were left open in an incubator for 5 min at 37 °C and 5% CO_2_ to level CO_2_ contents. Then pH was measured using a benchtop pH meter (Sartorius, Göttingen, Germany).

In order to evaluate the development of the pH during incubation, cells were cultivated in CCM, one group containing non-conditioned BG granules (0 h), four groups containing the BG being passivated for the designated amounts of time and a control group consisting of BMSCs in BG-free CCM. Supernatant of all groups was collected after 1 and 3 days and subjected to pH measurement as described above.

### 2.7. Visual Assessment of Cell Morphology and Growth Patterns

For the visual evaluation of cell morphology and growth patterns, BMSCs were stained with fluorescein diacetate (FDA). FDA can freely pass the cell membrane, then being intracellularly hydrolyzed to the green-fluorescent fluorescein by viable cells [24,25]. To achieve a better depiction of the BG granules and simultaneously visualize potentially remaining dead cells, propidium iodide (PI), which is not membrane-permeable and thus selectively intercalating into DNA of membrane-compromised cells [24,25], was used. After removal of supernatant 200 µL of staining solution including 8 µg/mL FDA (Sigma-Aldrich, Steinheim, Germany) and 20 µg/mL PI (Life Technologies, Darmstadt, Germany) were added to each well and incubated at 37 °C for 5 min. After being washed and kept in PBS, viability was analyzed using an Olympus IX-81 inverted fluorescence microscope (Olympus, Hamburg, Germany).

### 2.8. Quantification of Cell Viability

For quantitative assessment of cell viability three different BG concentrations, being 1, 2.5, and 5 mg/mL, were used to evaluate whether BG passivation has influence in a dose-dependent manner.

After removing CCM and washing cells in 100 µL PBS, 10 µg/mL FDA staining solution was added to the BMSCs. Culture plates were incubated for 5 min at 37 °C. Cells were washed in 100 µL PBS and thereafter lysed in 150 µL 0.5% Triton X-100 (Sigma-Aldrich) for 5 min. In a Wallac 1420 Victor microplate reader (Perkin Elmer, Waltham, MA, USA) fluorescence was detected at 535 nm.

### 2.9. Statistics

All groups consisted of five biological replicates. IBM SPSS Statistics (Version 25; IBM, Armonk, NY, USA) was used for statistical analysis. Passivated BG-groups were tested versus non-passivated BG and the control group using a paired *t*-test accepting *p* < 0.05 as the level of significance. Graphical design was performed with GraphPad Prism (Version 8.1.0; GraphPad Software, La Jolla, CA, USA). All values are shown as rounded means with standard deviation where applicable.

## 3. Results

### 3.1. The Impact of Passivation Periods on BG-Induced pH Alkalization of Cell Medium

**Figure 3:**
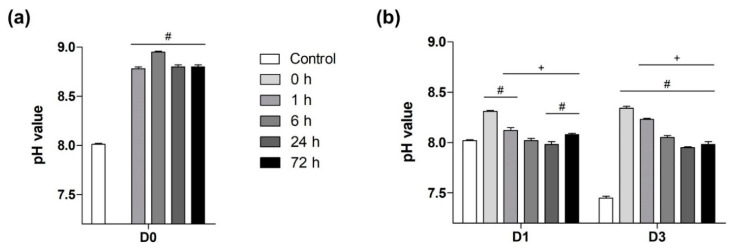
Evaluation of pH values. (**a**) pH values of the media used for preconditioning of the BG after 1, 6, 24, and 72 h in comparison to pure DMEM serving as control. Due to the missing passivation period, no pH values were obtained for the 0 h group since the BGs were directly introduced to the CCM without passivation. (**b**) pH values of all groups after 1 and 3 days in CCM. Values are displayed as means with standard deviation. Significant differences to the control group are indicated by (#), while (+) shows significant deviation to the non-passivated BG group.

The pH values measured in the media used for the passivation of 1 to 72 h were significantly higher than in the DMEM serving as control, that has not been in contact with BG (Figure 3a). At both time points in the cell culture setting, the highest pH values occurred in the group with non-passivated BG and decreased continuously with increasing duration of preconditioning except the 72 h-passivated group which was showing a minor increase (Figure 3b).

On D3, all BG groups showed significantly higher pH values than the control. On both days, the 24 h passivated group showed the same pH as the pure medium (control group) on D1 (Figure 3b).

### 3.2. Impact on Cell Morphology and Growth Patterns Particularly Showed during Early Incubation Period

**Figure 4:**
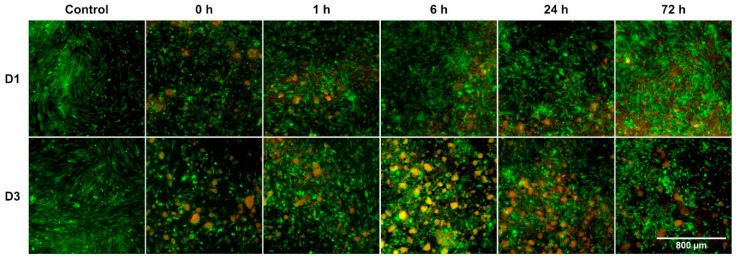
FDA/PI-staining presenting viable cells green-fluorescent and DNA of dead cells red-fluorescent. BG granules appear in orange color. Magnification: 40-fold, reference bar refers to 800 µm.

The effect of BG passivation had the most visible impact on D1 as could be observed in the microscopical analysis (Figure 3). While the physical presence of non-passivated 45S5-BG had the most negative influence on the consistent growth of BMSCs, cell density improved with increasing duration of preconditioning, being comparable to the control in the 72 h group. In the non-passivated BG group, cell density remained comparably low until D3, while cells cultivated with passivated BG grew faster. On D3, BMSCs in the passivated BG groups showed an increased adherence to the BG granules forming cell agglomerates. Nevertheless, BG presence led to lower cell density compared to the BG-free control (Figure 4).

Due to the performed washing steps detached dead cells could not be observed. However, PI staining led to an orange appearance of the BG granules (Figure 4).

### 3.3. Passivation Mediated Reduction of BG-Induced Cytotoxicity on All Days

**Figure 5:**
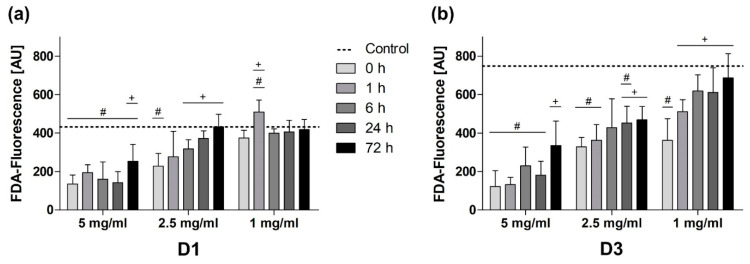
Quantification of cell viability at different BG concentrations on (**a**) D1 and (**b**) D3. Values are shown as means with standard deviation. Significant differences to the BG-free control group (shown as a dotted line) are indicated by (#) and significant differences to the non-passivated BG group are designated by (+).

The quantitative evaluation of cell viability showed different impacts of BG passivation depending on the used BG concentration (Figure 5). The viability of cells exposed to BG granules at a concentration of 5 mg/mL was significantly compromised at all times during the incubation period. Only BG passivation for 72 h caused a significant enhancement of viability compared to the non-passivated BG.

On both days at a concentration of 2.5 mg/mL, a preconditioning period of at least 24 h significantly improved viability compared to the non-passivated 45S5-BG group and cell viability was continuously improving with the duration of preconditioning (Figure 5).

At a concentration of 1 mg/mL no relevant advantages of BG passivation were found on D1, most likely due to a concentration-dependent lower overall cytotoxicity. On D3, an increasing viability was observed for longer preconditioning periods, showing significantly higher viability in all passivated BG groups compared to non-passivated BG (Figure 5).

## 4. Discussion

BGs increasingly gained in importance in the field of tissue engineering due to their many favorable properties, such as their proven ability to bond to hard and soft tissue [2,9,11,26]. When introduced to a static cell culture setting the high bioreactivity of BGs becomes a challenge due to the possible cytotoxic impact on cells [8]. While the dynamic conditions of an in-vivo setting permit the continuous dilution of ionic dissolution products, ions accumulate in a static cell culture setting leading to an excessive pH increase [27] that can be minimized through preconditioning approaches, e.g., by passivation of the BG prior to introduction to cell culture. Preconditioning of BG scaffolds has also been shown to enhance the scaffold in-vivo capacity of bone regeneration [28]. Nevertheless, this study focused on the aspects relevant for in-vitro settings necessary before progression to an animal model.

The analysis of the applied preconditioning procedures used over the last decades on the one hand demonstrated that the biocompatibility of BGs can be improved through passivation and on the other hand revealed the broad variety of utilized methods [8]. The available studies used individual settings concerning the choice of media and time used for the passivation, different shapes and compositions of BGs and the cell type exposed to the material, thus making it difficult to compare the efficiency of the used procedures. The range of the time periods scheduled for the passivation was broad, varying from 1 h to 24 days [8]. Therefore, to determine the most time- and cost-efficient approach, being as uncomplicated as possible while still ensuring a biocompatible cell culture setting, a direct comparison was inevitable.

In this study DMEM was chosen as preconditioning medium, as it is one of the most commonly used cell culture media [29]. Even though BG degradation dynamics may vary depending on the utilized medium [30], DMEM provides similar improvement of biocompatibility as solvents without organic components or simulated body fluids [17] and thus appears to be an appropriate choice for BG-passivation. The BG examined is the 45S5-BG composition, not only because it is well-characterized and often used but also because of its known cytotoxic effects in cell culture settings based on the high sodium content [13,16]. The cells used in this setting are human BMSCs, which exhibit favorable qualities for in-vitro and in-vivo BTE projects [9]. The periods of time determined for passivation are oriented towards the recommendations resulting from the literature, choosing 72 h as the maximum [8].

In this study all periods of passivation led to a reduction of pH-alkalization in the CCM and the BG induced cytotoxicity. The maximum pH observed in the non-passivated BG group almost reached a value of 8.4 clearly surpassing the physiological range. Longer passivation periods sustained more physiological pH values leveling the initial pH of 8, measured in the control group on D1, after a passivation period of 24 h. Regarding the absolute pH values it should be mentioned that the pH value of pure DMEM being 8 on D1 is relatively high. A slight elevation might be explained by the fact that cell culture occurred at the conventional 5% CO_2_ while DMEM is buffered to pH 7.4 at 10% CO_2_. Moreover, a benchtop pH-meter was used for pH evaluation at ambient air, shortly exposing the samples to comparably lower CO_2_ levels.

Between D1 and D3, the pH in the control group decreased—most likely due to acidic products of glycolytic cell metabolism [31]. Simultaneously, in the BG groups passivated for less than 24 h, the influence of metabolism was hidden by the alkalizing effect of the BG. Therefore, in the shortly passivated BG groups pH was further increasing between D1 and D3 while a passivation of at least 24 h could prevent a progression of pH-alkalization.

Cell viability was evaluated using an FDA-based fluorescence assay which was recently shown to directly correlate with cell number [32]. Therefore, absolute fluorescence intensities serve as direct correlates for both cell viability and number of viable cells. Alongside the changes in pH values, quantification of cell viability showed a positive tendency towards longer passivation periods at all concentrations. At the highest BG concentration, the cytotoxic impact of the BG was most pronounced only showing significantly higher cell viability in the 72 h passivated group. The lowest concentration, on the other hand, showed significantly higher viability for all passivated BGs compared to the non-passivated BG on D3, demonstrating that the passivation period needs to be adapted to the utilized BG concentration.

In this setting, a BG-concentration of 2.5 mg/mL was most suitable to evaluate the effects of BG preconditioning and is thus basis for the further discussion of the results. Here the BGs passivated for 24 h and 72 h showed significantly improved biocompatibility compared to the non-passivated BG on both days with the BGs passivated for 72 h showing the best properties in quantitative and qualitative analysis of cell viability. This might indicate that reducing pH to values around 8 is sufficient to provide a supporting environment for osteoblast precursor cells. Findings in literature are recommending different pH ranges as an optimum to improve cell proliferation and osteogenic differentiation. While Galow et al. demonstrated an enhanced proliferation and activity of osteoblasts at an alkalized pH in the range of 8.0–8.4 in-vitro [33], Monfoulet et al. stated an inhibition of osteogenic differentiation of BMSCs at pH values higher than 7.9 [34]. However, in our study BG-induced pH values below 8.1 were tolerated by BMSCs whereas more excessive alkalization led to increasing compromise of viability.

In the quantitative evaluation as well as the fluorescence microscopy, cell viability visibly increased in the control group between D1 and D3 as expected for the used culture setting [35,36]. In the meantime, for all BG groups viability remained at a similar level up to D3. Similar early cytostatic effects of BG extracts, attributed to a pronounced initial Ca^2+^ elution, were observed by Alcaide et al. [37]. This could mean, that despite passivation, cell viability is still impacted by the initial ion release of the BG, thus delaying the expected peak in cell proliferation due to a preceding stage of regeneration.

Furthermore, in the fluorescence microscopy it could be observed, that on D3 cells grew in agglomerates around passivated BG granules, especially in the 6 h and 24 h group. Preconditioning has been shown not to limit the HCA forming abilities of BGs [19]. In fact, the thickness of the HCA layer is seen to increase with longer period of contact with the medium [30,38,39]. It takes approximately 3 days for 45S5-BG to develop a HCA layer in DMEM [38]. On D3 of the culturing period different extents of HCA development can be expected, thus explaining a higher affinity of MSCs towards longer passivated granules.

A passivation period of more than 24 h did not show explicit advantages as passivation of the BGs for 24 h could prevent a strong pH alkalization. Nevertheless, cell viability was higher when BGs were passivated for 72 h despite the slightly higher pH. This advantage cannot be stated to pH alterations only but might be caused by the dynamic of pH alkalization that is disregarded when interpreting singular pH values. Especially the fast succession of pH alkalization may compromise cell metabolism and can cause enzyme alterations by influencing cell respiration [8,40]. Hence, passivation for 72 h is possibly providing a more stable milieu and better preventing rapid pH fluctuations. It should also be mentioned that ion release kinetics in the periphery and the inner region of BGs differ [41], thus making the measured pH values in the medium only a very indirect way to estimate the microenvironmental milieu at the BG/cell interface.

In this particular setting a passivation period of 24 h was sufficient to improve BG-biocompatibility. Nevertheless, the comparison of different BG concentrations showed that the required extent of passivation strongly depends on the used BG concentration [9,42]. Thus, the individuality of every setting makes a reassessment of adequate passivation periods inevitable. Further aspects impacting the primary biocompatibility of a setting are the BG-morphology (e.g., particle size and surface area) and -composition that are leading to altered ion release kinetics [16,18,19,37,43,44]. In this study, the concentration of ions released to the CCM has not been determined, as the dissolution characteristics of the 45S5-BG are well known [16,19,45,46,47]. An attractive possibility to evaluate the specific influence of ionic dissolution products is a setting including ICP-OES analysis and an indirect culturing approach, where cells are exposed to dissolution products instead of direct contact to the BG [15,16,48]. Especially when working with less well characterized BGs the suggested setting might further increase the understanding of BG-related cytotoxic effects.

When working with BGs demanding more extended passivation periods, renewing the medium during passivation might also be considered to better simulate physiological conditions [49]. As the main objective of this study was to distinguish a protocol that maximizes time-efficiency and involves the fewest possible work steps, we decided not to change the medium during the passivation periods.

It is also known that biomaterial-containing cell culture settings have a dissimilar impact on different cell types. A comparison of frequently used cell types in co-culture with 45S5-BG previously revealed that several osteoblast-like cell lines show high sensitivity to BG-induced cytotoxicity while other cells, namely primary human osteoblasts, BMSCs and the osteoblast-like cell line MG-63, presented as a suitable choice for BTE projects due to a higher tolerance towards cytotoxic effects [9]. According to these findings, there is no general solution for every particular setting. However, BG preconditioning by dynamic passivation in DMEM for 24 h following the protocol used in this study can be used as a guideline for planning a new experimental setting and can be adapted under consideration of the above-mentioned aspects.

## 5. Conclusions

The aim of this study was to compare BG passivation protocols and to find a suitable protocol for the use in static cell culture settings, that would minimize BG-provoked cytotoxicity and at the same time maximize time efficiency. The comparison of 4 different passivation periods of up to 72 h showed that the BG induced pH alkalization, that is held at least in parts responsible for cytotoxic effects on cells, was best reduced by a passivation of 24 h. Cell viability was continuously higher with longer periods of passivation revealing only a slight advantage for the 72 h passivated BGs over the BGs with 24 h of passivation. In accordance with these findings it can be assumed that BG passivation for 24 h for granules morphologically comparable to those used in this study is sufficient for static cell culture experiments. Our study also shows that dynamic passivation is an effective way to increase the compatibility of BGs for cell culture settings. Before introduction of BGs in cell culture, different passivation periods should be evaluated in order to meet the respective experimental settings (BG composition, BG morphology, cell types, etc.), e.g., by following the experimental protocols used in this study.

## Abbreviations

BG	bioactive glass
BMSC	bone marrow-derived stromal cell
BTE	bone tissue engineering
CCM	cell culture medium
CO_2_	carbon dioxide
DMEM	Dulbecco’s Modified Eagle’s Medium
FDA	fluorescein diacetate
HCA	hydroxycarbonate apatite
NEAA	non-essential amino acids
PBS	phosphate buffered saline
PI	propidium iodide
